# Next Generation Sequencing Reveals Skewing of the T and B Cell Receptor Repertoires in Patients with Wiskott–Aldrich Syndrome

**DOI:** 10.3389/fimmu.2014.00340

**Published:** 2014-07-18

**Authors:** Amy E. O’Connell, Stefano Volpi, Kerry Dobbs, Claudia Fiorini, Erdyni Tsitsikov, Helen de Boer, Isil B. Barlan, Jenny M. Despotovic, Francisco J. Espinosa-Rosales, I. Celine Hanson, Maria G. Kanariou, Roxana Martínez-Beckerat, Alvaro Mayorga-Sirera, Carmen Mejia-Carvajal, Nesrine Radwan, Aaron R. Weiss, Sung-Yun Pai, Yu Nee Lee, Luigi D. Notarangelo

**Affiliations:** ^1^Department of Immunology, Boston Children’s Hospital, Boston, MA, USA; ^2^Department of Hematology/Oncology, Boston Children’s Hospital, Boston, MA, USA; ^3^Department of Laboratory Medicine, Boston Children’s Hospital, Boston, MA, USA; ^4^Marmara University Medical Center, Istanbul, Turkey; ^5^Texas Children’s Hospital, Houston, TX, USA; ^6^Instituto Nacional de Pediatría, Mexico City, Mexico; ^7^Aghia Sophia Children’s Hospital, Athens, Greece; ^8^Department of Pediatric Hemato-Oncology, Hospital Mario Catarino Rivas, San Pedro Sula, Honduras; ^9^Centro de Neumologia y Alergia, San Pedro Sula, Honduras; ^10^Manati Medical Center, Manati, Puerto Rico, PR, USA; ^11^Ain Shams University, Cairo, Egypt; ^12^Maine Medical Center, Portland, ME, USA; ^13^Manton Center for Orphan Disease Research, Boston Children’s Hospital, Boston, MA, USA

**Keywords:** Wiskott–Aldrich syndrome, immune repertoire, next generation sequencing, deep sequencing, B cell receptor, T cell receptor, somatic hypermutation, clonotypic expansion

## Abstract

The Wiskott–Aldrich syndrome (WAS) is due to mutations of the *WAS* gene encoding for the cytoskeletal WAS protein, leading to abnormal downstream signaling from the T cell and B cell antigen receptors (TCR and BCR). We hypothesized that the impaired signaling through the TCR and BCR in WAS would subsequently lead to aberrations in the immune repertoire of WAS patients. Using next generation sequencing (NGS), the T cell receptor β and B cell immunoglobulin heavy chain (IGH) repertoires of eight patients with WAS and six controls were sequenced. Clonal expansions were identified within memory CD4^+^ cells as well as in total, naïve and memory CD8^+^ cells from WAS patients. In the B cell compartment, WAS patient IGH repertoires were also clonally expanded and showed skewed usage of *IGHV* and *IGHJ* genes, and increased usage of *IGHG* constant genes, compared with controls. To our knowledge, this is the first study that demonstrates significant abnormalities of the immune repertoire in WAS patients using NGS.

## Introduction

The Wiskott–Aldrich syndrome (WAS) is an X-linked disease characterized by the triad of eczema, microthrombocytopenia, and immunodeficiency (1, 2). Patients are also predisposed to autoimmunity and malignancy, resulting in poor clinical outcome overall (3, 4). The *WAS* gene encodes for the WAS protein (WASp) (5), which is expressed solely in hematopoietic cells, and is recruited to the inner cell membrane in response to activating signals, including engagement of the T and B cell antigen receptors (TCR and BCR) (6, 7). Upon activation, WASp recruits the Arp2/3 complex, triggering actin polymerization (8).

Deficiency of WASp is associated with significant immune abnormalities that affect all leukocytes (9). In particular, WAS patients manifest progressive T cell lymphopenia (10) and impaired formation of the immune synapse, defective IL-2 secretion, and reduced proliferation in response to TCR ligation (11, 12). The B cell compartment is also affected in WAS. Increased autoantibody production has been demonstrated in WASp-deficient patients and mice, and studies in mice lacking WASp solely in B lymphocytes have showed that this immune dysregulation reflects B cell intrinsic mechanisms, with increased hyper-responsiveness of WASp-deficient B cells to both BCR and toll-like receptor signaling (13, 14). WASp is also an important regulator of marginal zone (MZ) B cell maturation and positioning (15). It has been also reported that patients with WAS have an increased proportion of circulating CD19^+^ CD21^low^ CD38^low^ B cells (16, 17), which have been characterized as autoreactive-prone B cells (18). Finally, WAS patients have an increased number of peripheral transitional B cells and a concomitant decrease in immature B cells in the bone marrow (16). These abnormalities are probably secondary to decreased responsiveness to the chemotactic factor CXCL12, which signals through CXCR4 to retain immature B cells in the bone marrow.

T cell receptor- and BCR-mediated signaling plays a critical role in determining T and B cell fate during development and antigen-specific responses, and therefore, contributes to shaping the peripheral T and B cell repertoire. The diversity and complexity of the immune repertoire may in turn affect robustness of the immune response and disease outcome (19). Only limited information is available on TCR and BCR repertoire diversity and composition in WAS. Using complementarity determining region 3 (CDR3) spectratyping, Wada et al. demonstrated reduced diversity of the T cell receptor β (*TRB*) repertoire in WAS patients >15 years of age (20). More recently, limited diversity of the *TRB* repertoire was demonstrated with the same technique also in young WAS patients, and this abnormality was corrected by gene therapy (21). Finally, two groups have recently reported skewed usage of *IGHV* genes belonging to the *VH3* and *VH4* families in circulating B cells from patients with WAS, and reduced rate of somatic hypermutation (SHM) among Cγ- and Cα-containing immunoglobulin transcripts (16, 17). However, studies of T and B cell receptor repertoire diversity in WAS have been conducted using techniques (CDR3 spectratyping, targeted cloning, and sequencing) that permit only a descriptive assessment, or that sample only a limited number of sequences. Next generation sequencing (NGS) involves the use of high throughput sequencing technology to simultaneously amplify and analyze thousands of DNA or RNA sequences [reviewed in Ref. (22–25)]. Using this approach, single TCR and BCR rearranged genomic products or transcripts contained in a given sample can be amplified and individually sequenced. This permits robust analysis of repertoire diversity, and to assess the possible presence of clonotypic expansions; V, D, and J segment usage patterns; distribution and amino acid composition of CDR3 regions; sharing of CDR3 clonotypes between cell compartments; and SHM frequency. Here, we report for the first time on the use of NGS to analyze the expressed *TRB* and *IGH* repertoire of circulating T and B lymphocyte subsets isolated from patients with WAS and healthy controls. Our results demonstrate that patients with WAS present significant restriction of the *TRB* repertoire as well as abnormal distribution of the CDR3 length and skewed usage of V and J gene elements both at *TRB* and at the *IGH* loci. These abnormalities are present already at young age and are especially prominent within CD8^+^ T lymphocytes, possibly reflecting recurrent and/or chronic infections or the emergence of somatic revertant clones. Restriction of repertoire diversity may further contribute to the immunodeficiency of WAS.

## Materials and Methods

### Study subjects

Approval for the study was obtained from the Boston Children’s Hospital (BCH) institutional review board prior to initiation. Informed consent (and informed assent where appropriate) was granted by all study subjects and/or parents/guardians at the time of enrollment. Peripheral blood samples from patients with WAS (W1–W8) and healthy controls (C1–C6) were obtained by venipuncture either at BCH or at the collaborating institutions. Samples shipped from collaborators were processed within 4 days of the sample being drawn.

### Sample preparation

#### Isolating cell sub-populations

Peripheral blood mononuclear cells (PBMCs) were isolated from peripheral blood using Ficoll Paque Plus (GE Healthcare, Boston, MA, USA) gradient cell separation according to manufacturer’s instructions. Red blood cells were lysed using a 1× dilution of BD Pharm lyse (BD Biosciences) in sterile water and incubating the cells for 5–10 min. For total CD4, CD8, and B cell samples, isolated PBMCs were labeled sequentially with anti-human CD4 and CD8 magnetic beads (Miltenyi Biotec, San Diego, CA, USA) and respective fractions were obtained by positive magnetic selection while B cell-enriched fraction was obtained by negative selection of CD4^+^ and CD8^+^ PBMCs. For CD4 and CD8 naïve and memory populations, isolated PBMCs were labeled with mouse anti-human fluorescent antibodies: anti-CD3 PE/Cy7, anti-CD4 PE, anti-CD8 APC, anti-CD45RA FITC, and anti-CCR7 Pacific Blue (eBiosciences, San Diego, CA, USA). Cells were stained for 30 min and then washed and sorted on a BD FACS Aria II cell sorter. Naïve CD4^+^ and CD8^+^ T cells were sorted based on the CD45RA^+^ CCR7^+^ phenotype. With this sorting strategy, the naïve T cell compartment did not include CD45RA^+^ CCR7^−^ cells that correspond to the exhausted effector memory T cell (T_EMRA_), which is often expanded in patients with WAS (Table 1). Cell purity was checked after sorting and was consistently >92%.

**Table 1:** **Patient characteristics**.

ID	W1	W2	W3	W4	W5	W6	W7	W8
Age	54 years	14 months	3 years	10 months	23 months	10 years	11 months	19 months
Infections	Pneumonia, otitis	PCP pneumonia	EBV, CMV, RSV, HSV stomatitis	Orchitis	Ocular HSV	Otitis, bronchitis, pneumonia	Cellulitis	Febrile illnesses
Mutation	IVS6 + 5,G > A	c. 454 C > T (p.Q152X)	c.35delAA (p.G12fsX)	c.8997_9001 dup TACTC (p.110fsX13)	c.1154delG (p.G378AX84)	IVS8 + 1,G > A	c.1116 del T	IVS3 − 2,A > G
WASp expression	CD8^+^ T cells (revertant)	None	CD8^+^ T, NK cells (all revertants)	None	None	CD4^+^, CD8^+^ T cells (revertants)	None	Reduced
CD3^+^ (cells/μL)	705 (1000–2600)	1803 (1900–6200)	1766 (1400–6200)	1054 (1900–6200)	1522 (1900–6200)	2986 (1000–2600)	1703 (1900–6200)	104 (1900–6200)
CD4^+^ (cells/μL)	611 (530–1500)	1533 (1300–3400)	890 (700–2200)	897 (1400–4300)	152 (1300–3400)	1078 (530–1500)	1377 (1400–4300)	1142 (1300–3400)
Naïve CD4^+^ (%)	36 (21–61.4)	59.9 (66.3–89.4)	70.8 (65.2–84.8)	74.5 (76.7–91.4)	ND	11.6 (57.4–84.9)	74 (76.7–91.4)	67.7 (66.3–89.4)
Effector mem. CD4^+^ (%)	30 (7.6–25.1)	16.8 (1.2–9.4)	12.9 (2.9–9.8)	ND	ND	76.2 (3.3–15.2)	9.1 (1.1–5.3)	11.7 (1.3–9.4)
Central mem. CD4^+^ (%)	32.1 (26.8–62.1)	21.5 (9.2–22.4)	13.9 (10.5–23.2)	ND	ND	9.3 (11.3–26.7)	15.9 (6.7–15.6)	18.7 (9.2–22.4)
CD8^+^ (cells/μL)	92 (330–1100)	235 (620–2000)	660 (490–1300)	94 (500–1700)	1309 (620–2000)	1669 (330–1100)	250 (500–1700)	277 (620–2000)
Naïve CD8^+^ (%)	9.7 (11.4–66.5)	53.6 (57.8–82.9)	14.1 (39–89)	ND	ND	1.9 (28.4–80.6)	59.3 (62.1–94)	38.1 (57.8–82.9)
Effector mem. CD8^+^ (%)	45.4 (16.8–54.6)	24.7 (5.1–25.1)	26.8 (3.4–28.2)	ND	ND	76.7 (6.2–29.3)	20.7 (1.3–19.5)	37.7 (5.1–25.1)
Central mem. CD8^+^ (%)	12.6 (3.7–23.2)	1.8 (1.7–8.5)	0.6 (0.9–5.7)	ND	ND	0.8 (1–4.5)	2.6 (0.9–5.6)	1.5 (1.7–8.5)
T_EMRA_ (%)	32.3 (5.6–43.9)	19.9 (6.4–20.8)	58.5 (4.8–30)	ND	ND	20.7 (9.1–49.1)	17.5 (1.5–22.7)	22.7 (6.4–20.8)
CD19 (cells/μL)	174 (110–570)	980 (60–2600)	282 (390–1400)	1265 (610–2600)	ND	400 (270–860)	1285 (610–2600)	497 (610–2600)
CD38^hi^ CD21^lo^ (%)	19 (2.2–13.3)	13.6 (10.3–30.4)	12.5 (7.4–23.7)	ND	ND	ND	19 (9.4–27)	9.6 (10.3–30.4)
CD38^lo^ CD21^+^ (%)	60	69	44.2	ND	ND	66.4	60.5	71.6
IgD^+^ CD27^+^ (%)	0.4 (7–23.8)	4.4 (3–10.7)	1.2 (2.7–19.8)	ND	ND	5.2 (5.2–20.4)	2.7 (3–10.7)	1.2 (2.7–19,8)
IgD^−^CD27^+^ (%)	7 (8.3–27.8)	4.4 (1.4–11.9)	4.6 (3.3–7.4)	ND	ND	11.3 (10.9–30.4)	5.7 (1.4–11.9)	3.4 (3.9–13.6)
CD38^hi^ CD24^lo^ (%)	4.4 (0.1–2.4)	2.2 (0.1–12.6)	2.4 (0.2–4.4)	ND	ND	ND	2.1(0.2–3.5)	1.2 (4.1–13.9)
CD21^lo^ CD38l^o^ (%)	6.8 (1.1–10.7)	5.3 (1.6–7.6)	29.9 (2.3–9.3)	ND	ND	ND	8 (0.9–5.4)	10.1 (1.6–7.6)
IgG (mg/dL)	968 (639–1344)	410 (400–1300)	864* (600–1500)	924* (300–1500)	ND	1048* (630–1344)	898* (300–1500)	1292* (400–1300)
IgA (mg/dL)	763 (70–312)	59 (20–230)	128 (50–150)	71 (16–100)	ND	511 (70–312)	82 (16–100)	496 (20–230)
IgM (mg/dL)	15 (34–210)	13 (30–120)	<5 (22–100)	33(25–115)	ND	21 (34–210)	59 (25–115)	<5 (30–120)
IgE (kU/L)	238 (<200)	95 (<30)	1914 (<200)	128 (<30)	ND	5769 (<200)	370 (<30)	170 (<30)

*Clinical data for 8 WAS patients (W1–W8). ND, study not done; IVIG, intravenous immunoglobulins. B cell subsets were defined as follows: CD19^+^ CD38^hi^ CD21^lo^, transitional B cells; CD19^+^ CD38^lo^ CD21^+^, naïve B cells; CD19^+^ IgD^+^ CD27^+^, unswitched memory B cells; CD19^+^ IgD^−^ CD27^+^, switched memory B cells; CD19^+^ CD38^hi^ CD24^lo^, plasmablasts*.

*On IVIG at the time of assessment.

#### Reverse transcription PCR and sequencing

Sorted cells were placed in Trizol (Ambion, Inc/Life Technologies, Grand Island, NY, USA) and mRNA was extracted according to the manufacturer’s instructions. mRNA samples were then subjected to reverse transcription PCR (RT-PCR) using a Qiagen OneStep RT-PCR kit (Qiagen Inc., Valencia, CA, USA) and iRepertoire^®^ human T cell beta receptor (HTBR) primers (iRepertoire Inc., Huntsville, AL, USA) for T cell samples, or human immunoglobulin heavy chain (HIGH) primers for B cell samples, under the reaction conditions specified by iRepertoire^®^. Each primer contained a barcode that was integrated into the PCR, allowing donor identification. A second PCR was then carried out using a Qiagen Multiplex PCR kit and the iRepertoire^®^ 454 Lib-A primers, again under conditions specified by iRepertoire^®^. The samples were run on a 2% agarose gel, and then the DNA in the 350–500 bp range was excised and extracted from the gel using a Qiagen Gel Extraction kit, according to manufacturer’s instructions. The samples were then gel-purified second time in order to further eliminate primers and non-specific amplification from the samples. Finally, the DNA content of the samples was assessed using the Flash Gel System (Lonza, Hopkinton, MA, USA) and PicoGreen (Invitrogen, Inc/Life Technologies, Grand Island, NY, USA) quantification system to determine qualitative and quantitative concentration of the PCR products. Pooled sample libraries were then sequenced using the GS Junior 454 platform (Roche, Mannheim, Germany).

### Analysis of sequencing data

iRepertoire^®^ provided raw data on V, D, and J segment usage for T and B cell samples and C-region usage for B cell samples. They also provided filtered DNA sequences utilized for additional analyses. Information on CDR3 length was obtained from these filtered sequence files. Unique filtered DNA sequences were also multiplied to reflect the number of copies present for each sequence in a population using Microsoft excel. CSV output files from Excel were then converted into FASTA format using Geneious software. FASTA files were uploaded to the ImMunoGeneTics (IGMT) database and the IMGT/high V-Quest web-based analysis tool (26), which provided sequence output files. The IGMT mutation analysis files were used to calculate the number of mutations at each amino acid residue for the various CDR and framework regions (FR). The mutation index was calculated by dividing the total number of mutations in each FR or CDR region, by the total nucleotide length of the FR or CDR region analyzed, and finally dividing that by the total number of unique sequences obtained for each population analyzed. IGMT output files were uploaded into IgAT analysis tool (27) to allow analysis of biodiversity. Finally, rarefaction curves for each sample were generating using PAST (28).

### Statistical analyses

As outcome variables were ordinal in nature and a normal distribution could not be assumed for any of the dependent variables, the Mann–Whitney test was used to assess for differences between controls and WAS patients. Analysis was performed using PRISM version 6 (Graph Pad).

## Results

### Clinical and laboratory features

Eight WAS patients (age range: 10 months to 54 years; median: 21 months) and 6 healthy controls (age range: 9 months to 5 years; median 4 years) were included in the study. None of the patients had received hematopoietic cell transplantation (HCT) or gene therapy at the time of the study. The clinical, immunological, and molecular features of WAS patients are reported in Table 1. A history of recurrent infections was documented in seven of the eight WAS patients, and one of them (W3) had chronic viral infections. None of the patients had significant autoimmunity. WASp expression was analyzed in all patients (data not shown). Four patients lacked WASp expression in all blood lineages (W2, W4, W5, and W7); one patient (W8) had residual, but reduced, protein expression. Three patients (W1, W3, and W6) had somatic reversions allowing WASp expression in CD8^+^ T cells only (W1), CD8^+^ and NK lymphocytes (W3), or in CD4^+^ and CD8^+^ cells (W6). Immunological abnormalities detected in patients included a variable degree of T cell lymphopenia, accumulation of effector memory and of CD8^+^ T_EMRA_ cells, a low number of unswitched memory B cells, and an increased proportion of CD19^+^ CD21^low^ CD38^low^ B cells (Table 1). Immunoglobulin serum levels were tested in seven patients; low IgM were observed in five patients, and increased IgA in three. Finally, all seven patients tested had elevated serum IgE (Table 1).

### Sequencing output and quality analysis

The mean number of reads obtained for all samples was 7,080 (median: 4,631; range 114–31,731). No reads were obtained for CD4^+^ cells from patients W4 and W5, due to an error in sample processing; these samples were therefore excluded from all analyses. The richness of each sample’s data was determined by rarefaction curves, which measure increase of diversity along the depth of sequencing. Rarefaction curves plateau as the vast majority of species present in a population have been sampled. Rarefaction curves indicated thorough capturing of unique *TRB* sequences for most of the T cell subsets analyzed (data not shown). Similar results were also obtained for *IGH* repertoire (data not shown).

### Analysis of the *TRB* repertoire demonstrates clonotypic expansions and skewed usage of V and J segments in CD8^+^ lymphocytes from patients with WAS

**Figure 1:**
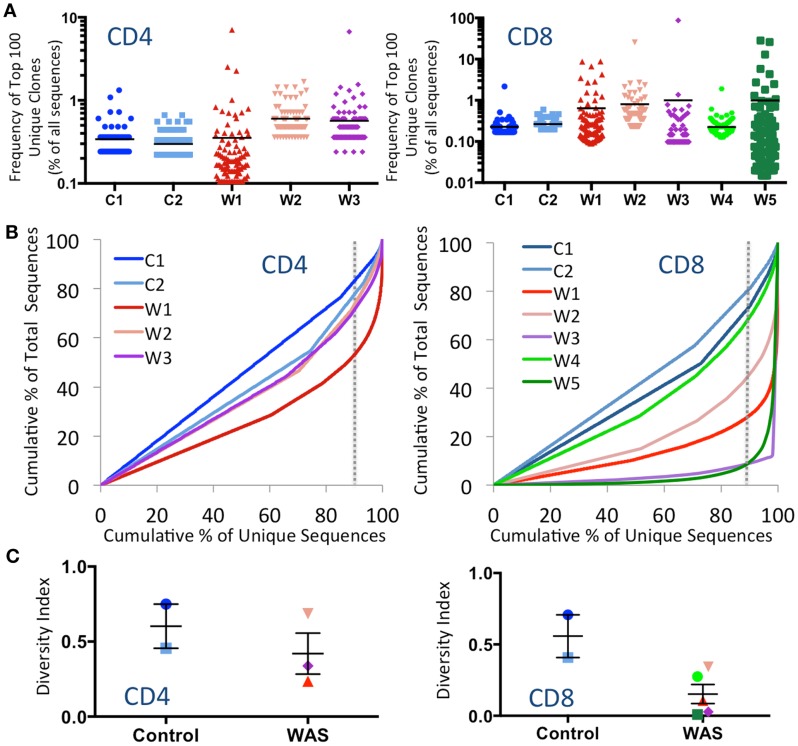
**Clonal expansions and diversity of the CD4^+^ and CD8^+^ TRB repertoire**. **(A)** The relative frequency of the 100 most abundant clones is displayed as a proportion of the total sequences for both CD4^+^ (left panel) and CD8^+^ (right panel) populations. The black line indicates the mean of the sample. **(B)** The degree of clonal expansion for CD4^+^ and CD8^+^ populations is displayed by plotting the cumulative percentage of unique clones (*x*) vs. the cumulative percentage of total sequences (*y*). A slope of one would indicate an even distribution of clonotypes. The dotted gray vertical line indicates 90% of cumulative unique sequences, with sequences to the right of the line corresponding to the top 10% most abundant unique clonotypes. **(C)** The diversity of each patient’s repertoire was determined by dividing the number of unique sequences over the total sequences for CD4^+^ and CD8^+^ populations. Bars represent mean and SE. In all panels, the same color is used to identify individual control subjects and patients.

To investigate whether CD4^+^ and CD8^+^ cells from WAS patients contained expanded clonotypes, the frequency of the top 100 most abundant unique clonotypes was expressed as a percentage of the total number of sequences obtained. Clonotypic expansions were identified among CD4^+^ lymphocytes from patients W2 and W3 and among CD8^+^ cells from patients W1, W2, W3, and W5 (Figure 1A). To investigate evenness of clonotype size distribution, we plotted the cumulative percentage of total TRB amino acid repertoire vs. the cumulative percentage of unique TRB amino acid clonotypes ordered by increasing clonotype size. In this representation, an even clonotype size distribution (i.e., all clonotypes being of the same size) would be represented by the bisector line, and the extent of deviation from the bisector would indicate the unevenness of the distribution. As shown in Figure 1B, clonotypic expansions were demonstrated for CD4^+^ cells from patient W1, and for CD8^+^ cells from patients W1, W2, W3, and W5. In particular, the top 10% most abundant unique clones accounted for <20% of the CD4^+^ total sequences and <30% of the CD8^+^ total sequences in control subjects. In contrast, the top 10% most abundant unique sequences accounted for as many as 50% of the CD4^+^ total sequences in patient W1 and more than 90% of the CD8^+^ total sequences in patients W3 and W5.

To measure diversity, the number of unique clones in each sample was divided by the total number of sequences obtained. A diversity index of 1 indicates that each unique clone is represented only one time, whereas diversity approaching 0 indicates that only a few clones were redundantly expressed. As shown in Figure 1C, the diversity of WAS CD4^+^ cells was variable, but overall similar to that of CD4^+^ cells from healthy controls. In contrast, CD8^+^ cells from WAS patients had markedly reduced diversity, although statistical significance was not reached, due to the limited sample size.

**Figure 2:**
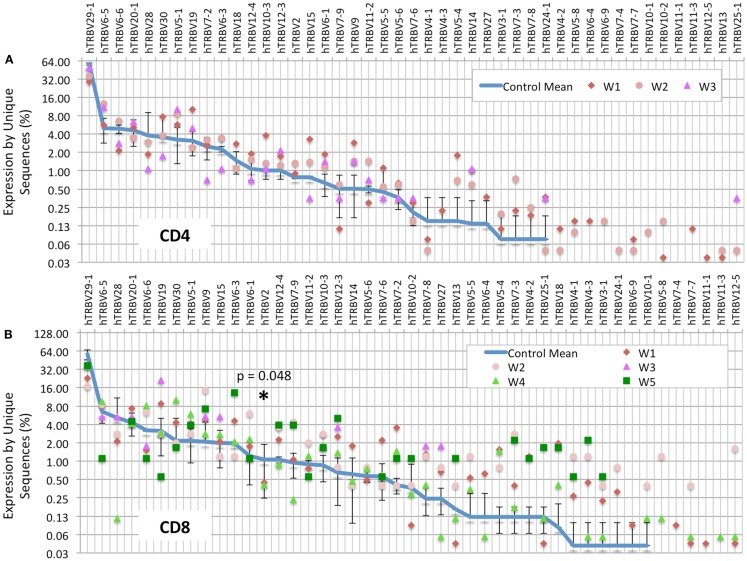
***TRBV* gene usage is skewed in the CD8^+^ population of WAS patients**. The frequency of usage of individual *TRBV* genes among unique *TRB* sequences is displayed for the CD4^+^ **(A)** or CD8^+^ **(B)** cells. The blue line indicates the average frequency of the two control subjects, and the error bars indicate the SD of the frequency of the controls. *p* Values were calculated using Mann–Whitney test between controls and WAS for each V segment. *p < 0.05.

Analysis of the frequency of usage of individual *TRBV* genes among unique CD4^+^ clonotypes revealed a similar pattern in WAS patients and controls (Figure 2A). In contrast, for CD8^+^ cells, there was a wider distribution in the frequency of *TRBV* segment usage in unique CD8^+^ clonotypes from WAS patients compared with controls (Figure 2B). In particular, *TRBV2* was less frequently expressed in CD8^+^ cells from WAS patients than controls (*p* = 0.05). A few other genes (*TRBV29-1, TRBV9, TRBV10-3*, and *TRBV12-3*) tended to be under- or hyper-represented in CD8^+^ T cells from patients vs. controls, but the trends did not reach significance. It is important to note that V gene results are displayed on a logarithmic graph, so while values were obtained for all WAS patients for each gene, genes with expression approaching 0 will fall below the lower limit of the *y*-axis on these graphs, and therefore, not be displayed.

**Figure 3:**
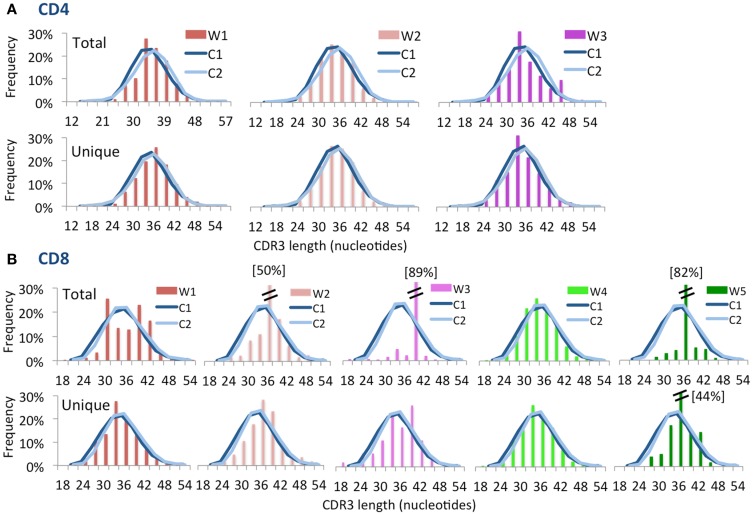
**Complementarity determining region 3 length distribution of unique and total sequences for CD4^+^ and CD8^+^ TRB repertoire**. Distribution of CDR3 nucleotide length frequencies of CD4^+^ **(A)** and CD8^+^ **(B)** populations for both total and unique clones for each WAS patient compared to control subjects C1 and C2 (shown as curves). A double bar indicates the value of the bar has exceeded the axis limit, and the maximum value is indicated within brackets.

Computational analysis permits to construct “virtual spectratyping” to indicate the relative frequency with which sequences of CDR3 region of the *TRB* (CDR-B3) of various lengths are represented among both the total and the unique *TRB* sequences. A similar distribution of the CDR-B3 length was observed for both unique and total sequences obtained from CD4^+^ cells from patients and controls (Figure 3A). In contrast, significant deviation from the bell-shaped curve was observed for total CDR-B3 sequences from CD8^+^ cells from patients W1, W3, and W5 (Figure 3B), and aberrant kurtosis was detected in patient W2 (Figure 3B). Furthermore, an abnormal distribution of CDR-B3 length was also detected for unique sequences obtained from CD8^+^ cells from patients W3 and W5. Overall, these data confirm that skewing of the *TRB* repertoire is especially prominent in CD8^+^ cells from patients with WAS. Discrepancy in virtual spectratyping between unique and total sequences obtained from CD8^+^ cells from patients W1 and W2 is consistent with the uneven distribution of clonotype sizes in these patients (Figure 1B). However, abnormalities of virtual spectratyping among unique sequences from CD8^+^ lymphocytes from patients W3 and W5 is indicative of markedly reduced diversity of the *TRB* repertoire, as also shown in Figure 1C.

**Figure 4:**
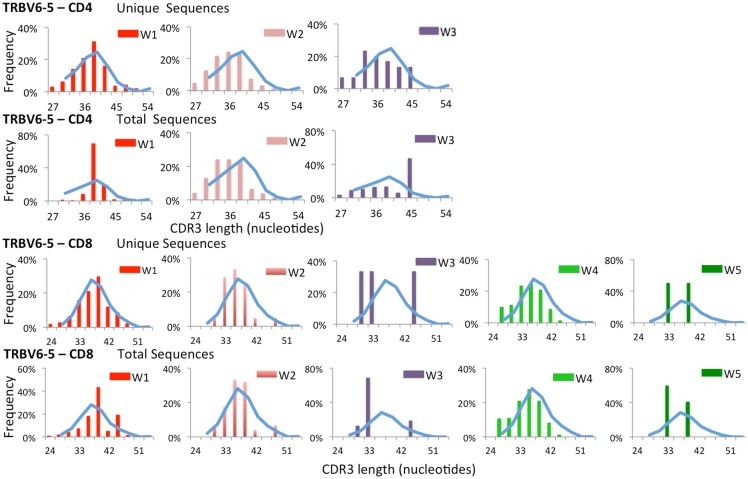
**Complementarity determining region 3 length analysis of *TRBV6-5 expressing clonotypes* subgroups**. Length distribution of the CDR3 region of unique and total clones containing *TRBV6-5* in both CD4 and CD8 populations from WAS patients (bars) and controls (blue line).

To further confirm that virtual spectratyping may identify specific abnormalities of the immune repertoire, we analyzed distribution of CDR-B3 length among unique and total sequences containing *TRBV6-5*, a gene that was abundantly used both in patients and controls (Figure 2). As shown in Figure 4, frequency of CDR-B3 transcripts of various lengths followed a bell-shaped pattern among both unique and total sequences from CD4^+^ and CD8^+^ lymphocytes from healthy controls (blue line). A similar pattern was also observed for unique CD4^+^ and CD8^+^ clonotypes from patient W1. In contrast, an aberrant distribution of CDR-B3 length was detected among total sequences from CD4^+^ and CD8^+^ lymphocytes of the same patient, consistent with clonotypic expansions, previously shown in Figure 1B. On the other hand, skewing of CDR-B3 distribution was observed among both unique and total CD8^+^ sequences from patients W3 and W5, indicative of severe repertoire restriction. Indeed, only three unique *TRBV6-5*-containing sequences were detected in CD8^+^ lymphocytes from patient W3, and two such sequences in patient W5. Overall, these data demonstrate the analytical power of NGS in revealing abnormalities of the immune repertoire, and confirm that the *TRB* repertoire of WAS patients is characterized by both reduced diversity and clonotypic expansions.

**Figure 5:**
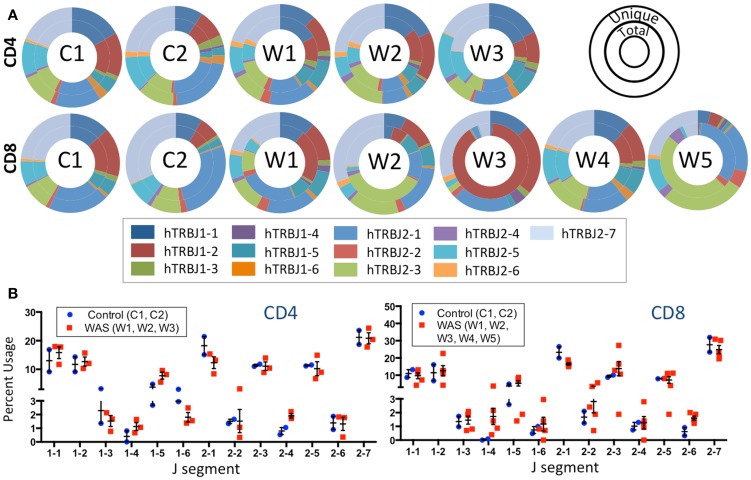
**J segment usage is skewed in the TRB repertoire of CD8^+^ cells in WAS patients**. **(A)** Donut graphs compare the representative frequencies of *TRBJ* segment usage for total (inner ring) and unique (outer ring) sequences for controls and WAS patients (see also inset key). **(B)** Direct comparison of J segment usage between WAS patients and controls.

The frequency of *TRBJ* genes usage was also analyzed for both CD4^+^ and CD8^+^ lymphocytes of patients and controls, and results for both total (inner ring) and unique (outer ring) sequences were plotted using Microsoft donut graphical representation (Figure 5A). With this function, clonotypic expansions manifest as significant differences in the pattern of outer vs. inner ring. Minor differences were observed in usage of *TRBJ* segments between total and unique clones in CD4^+^ cells from WAS patients. In contrast, remarkable differences in the distribution of *TRBJ* gene usage between unique vs. total sequences were observed for CD8^+^ cells from WAS patients W1, W2, W3, and W5 (Figure 5A), confirming the presence of clonotypic expansions in this population. Analysis of the frequency of *TRBJ* segment usage showed that WAS patients had decreased usage of *TRBJ2-1* (among both CD4^+^ and CD8^+^ cells) and an increased usage of *TRBJ2-6* among CD8^+^ lymphocytes (Figure 5B).

### Analysis of *TRB* repertoire in naïve and memory T cells reveals a variable pattern of uneven clonotype size distribution in patients with WAS

**Figure 6:**
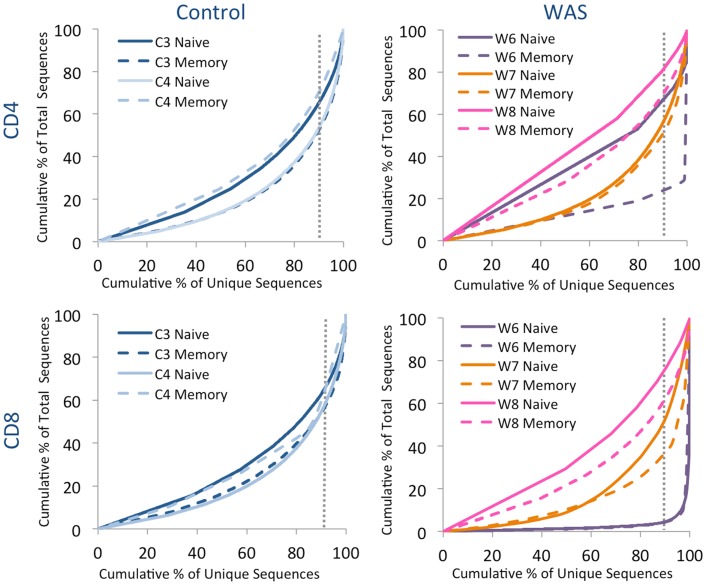
**Clonal expansion of the *TRB* repertoire in CD4^+^ and CD8^+^ naïve and memory lymphocytes**. The degree of clonal expansion for each patient’s repertoire is displayed by plotting the cumulative percentage of unique sequences (*x*) vs. the cumulative percentage of total sequences (*y*) in controls (left panels) and patients (right panels). A slope of one would indicate an even distribution of clonotypes. The dotted gray vertical line indicates 90% of cumulative unique sequences, with sequences to the right of the line corresponding to the top 10% most abundant clonotypes.

Analysis of the distribution of cumulative percentages of unique and total sequences demonstrated a similar pattern for naïve and memory CD4^+^ and CD8^+^ cells from controls, with the top 10% most abundant unique clones accounting for 35–50% of the total clones (Figure 6). In contrast, clonotypic expansions were identified in memory CD4^+^ cells from patient W6 (where the top 10% unique sequences accounted for more than 75% of total sequences), and in both naïve and memory CD8^+^ cells from the same patient, with the top 10% most abundant unique clonotypes accounting for over 90% of the total sequences (Figure 6). Uneven distribution of clonotype size (albeit not as pronounced as for patient W6) was also observed for naïve and memory CD8^+^ cells from patient W7.

**Figure 7:**
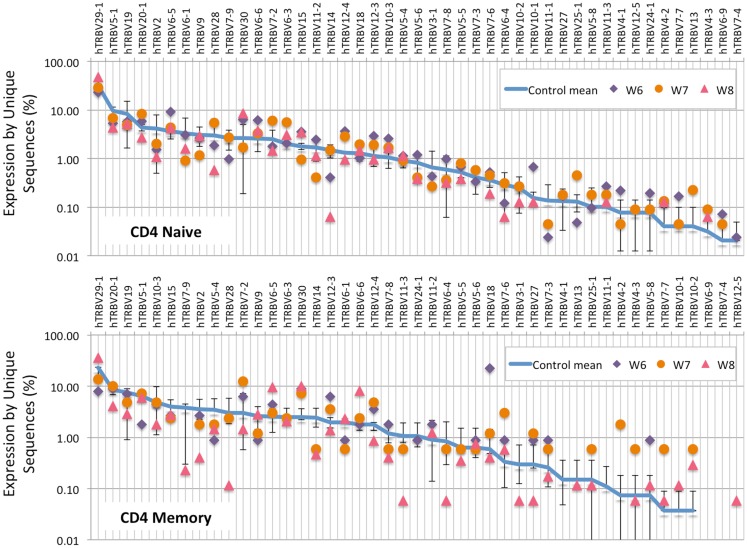
***TRBV* gene usage in CD4^+^ naive and memory lymphocytes from healthy controls and patients with WAS**. The frequency of usage of individual *TRBV* genes in unique *TRB* sequences is displayed for CD4^+^ naïve (top panel) or memory (bottom panel) lymphocytes. The blue line indicates the average frequency of the two control patients, and the error bars indicate the SD of the frequency of the controls.

**Figure 8:**
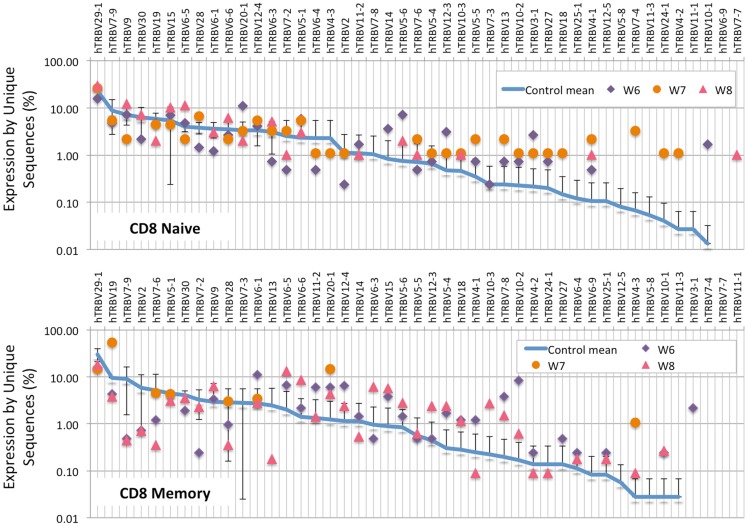
***TRBV* gene in CD8^+^ naive and memory lymphocytes from healthy controls and patients with WAS**. The frequency of usage of individual *TRBV* genes in unique *TRB* sequences is displayed for CD8^+^ naïve (top panel) or memory (bottom panel) lymphocytes. The blue line indicates the average frequency of the two control patients, and the error bars indicate the SD of the frequency of the controls.

Analysis of the frequency of *TRBV* segment usage for all unique sequences revealed a similar pattern in naïve CD4^+^ lymphocytes from patients and controls (Figure 7, top panel). However, a broader distribution was observed in *TRBV* gene usage among memory CD4^+^ cells from patients with WAS (Figure 7, lower panel), with increased usage of the *TRBV30* and reduced usage of the *TRBV7-9* genes as compared to controls. A similar analysis, performed on CD8^+^ lymphocytes, showed that naïve CD8^+^ cells from WAS patients had a tendency toward increased usage of *TRBV* genes that are rarely utilized in healthy controls (Figure 8, top panel). A broad distribution of *TRBV* gene usage was observed among memory CD8^+^ cells from patients with WAS (Figure 8, lower panel). In order to investigate further usage of *TRBV* genes in WAS, the *TRBV* genes were grouped into a D-proximal set (from *TRBV30* to *TRBV27*) and a D-distal set (from *TRBV25-1* to *TRBV2-1*). Increased usage of D-distal *TRBV* genes was demonstrated in CD8^+^ lymphocytes (and in particular, among memory CD8^+^ cells) from patients with WAS vs. controls (*p* < 0.05; data not shown). Altogether, these data confirm that abnormalities of the *TRB* repertoire in WAS are more often observed in the CD8^+^ and in memory T cell compartments.

### Analysis of the *IGH* repertoire reveals clonotypic expansions and skewed usage of *IGHV* and *IGHJ* segments in peripheral B cells from patients with WAS

**Figure 9:**
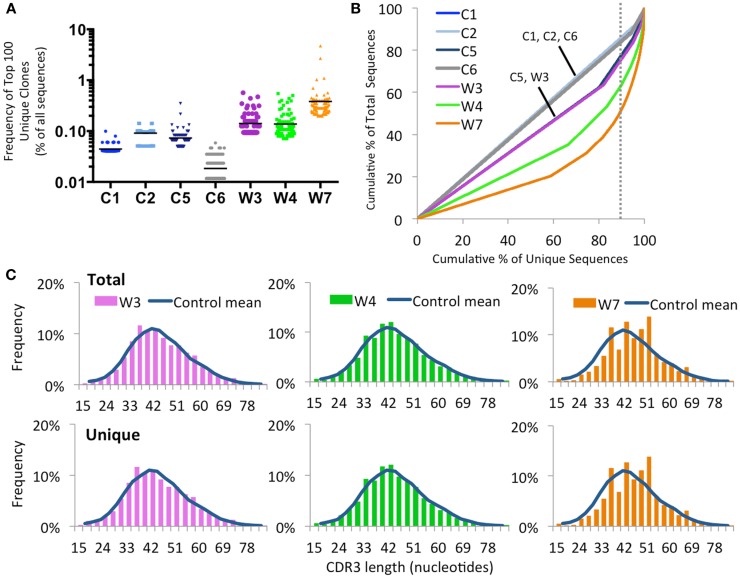
**Clonal expansion of the IGH repertoire in circulating CD19^+^ lymphocytes**. **(A)** The relative frequency of the 100 most abundant clones is displayed as a proportion of the total sequences in four healthy controls (C1–C4) and three WAS patients (W3, W4, and W7). The black line indicates the mean of the sample. **(B)** The degree of clonal expansion for each patient’s repertoire is displayed by plotting the cumulative percentage of unique clones (*x*) vs. the cumulative percentage of total sequences (*y*). A slope of one would indicate an even distribution of clonotypes. The dotted gray vertical line indicates 90% of cumulative unique sequences, with sequences to the right of the line corresponding to the top 10% most abundant clonotypes. **(C)** Distribution of CDR3 nucleotide length frequencies for both total and unique sequences in individual WAS patients compared to the mean in controls C1 and C2 (shown as a curve).

As shown in Figure 9A, there was a trend for the mean frequency of the top 100 most abundant unique *IGH* clonotypes to be higher in patients with WAS than in controls. The uneven distribution of clonotype size was confirmed when plotting the cumulative percentage of unique vs. total sequences (Figure 9B). Furthermore, virtual spectratyping of the CDR3 region of *IGH* transcripts (CDR-H3) demonstrated deviation from the bell-shaped pattern for both total and unique *IGH* transcripts from patient W7, with presence of a large fraction of clonotypes with longer CDR-H3 length (51 nucleotides) (Figure 9C).

**Figure 10:**
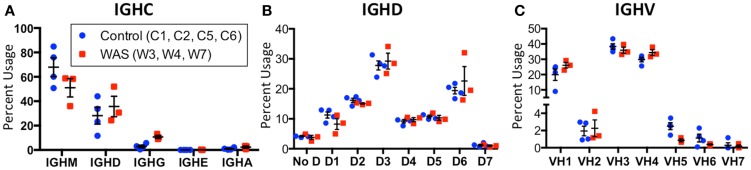
**Usage of *IGHV, IGHD*, and *IGHC* families in circulating B cells from controls and WAS patients**. Percent usage of *V* **(A)**, *D* **(B)**, and constant **(C)** segment families in unique *IGH* clonotypes is shown for both controls (blue dots) and WAS patients (red squares). Bars indicate the mean and SE.

**Figure 11:**
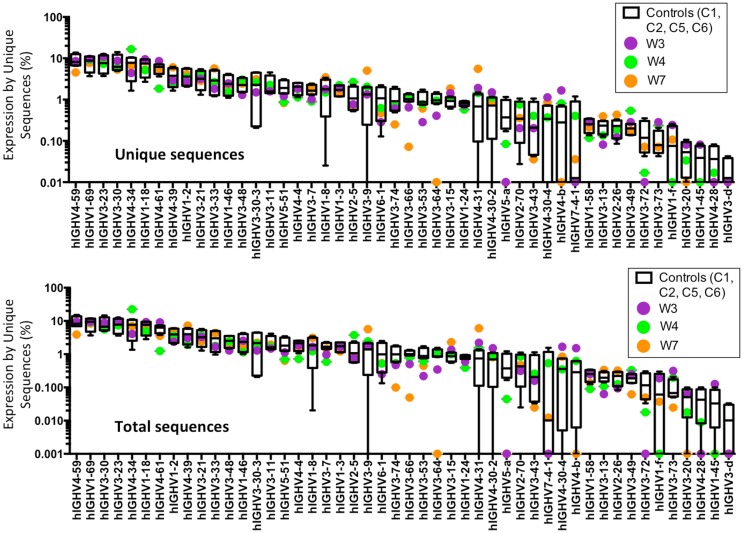
**Individual *IGHV* gene usage in circulating CD19^+^ cells from WAS patients and controls**. The frequency of usage of individual *IGHV* genes in unique *IGH* sequences is displayed. Boxes indicate the high, low, and median values, and error bars indicate the 5th and 95th percent confidence intervals for controls. WAS patients are identified by filled symbols.

An increased frequency of γ-containing *IGH* transcripts was detected within unique and total sequences from patients with WAS, associated with a trend toward reduced frequency of μ-containing transcripts (Figure 10A). Overall, μ-containing transcripts were most predominantly expressed both in controls and WAS patients. No differences were observed in the frequency of usage of *IGHD* genes (Figure 10B). When usage of *IGHV* families was analyzed, patients with WAS showed decreased usage of *IGHV5* (Figure 10C). Overall, the pattern of *IGHV* gene usage by unique and total sequences was similar in patients and controls (Figure 11), indicating a minimal effect from clonotypic expansions in the B cell repertoire. However, there was a tendency toward decreased usage of *VH3-66, VH5-51, VH3-74, VH3-53*, and *VH3-72*, whereas *VH3-9, VH3-15, VH4-31, VH4-30-2*, and *VH4-30-4* tended to be overexpressed in WAS patients compared with controls. One patient (W4) had an increase in *VH4-34* expression.

**Figure 12:**
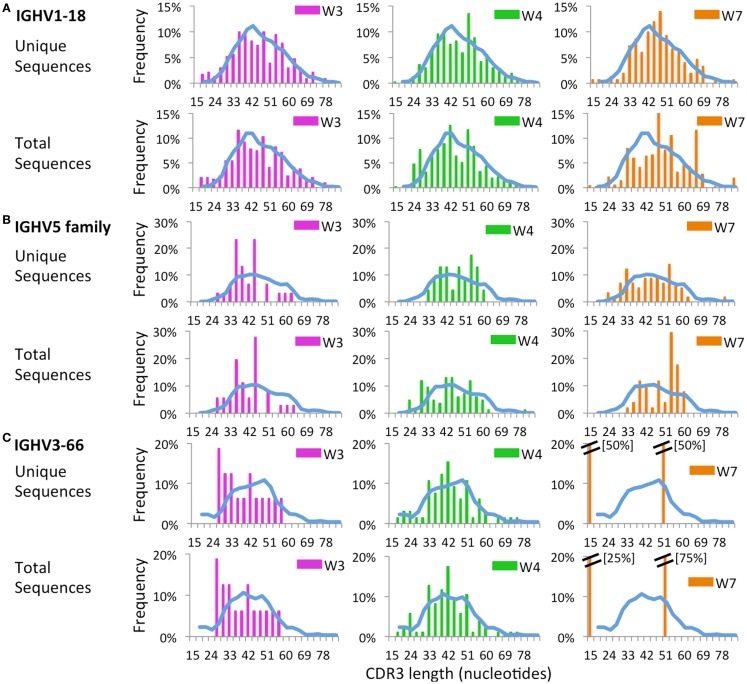
**Complementarity determining region 3 length analysis of *IGHV* subgroups**. CDR3 length analysis was performed on CD19^+^ cells expressing *IGHV1-18* **(A)**, *IGHV5* **(B)**, or *IGHV3-66* **(C)**. The mean distribution of controls (C1 and C2) is displayed as a blue bar. A double bar indicates the value of the bar has exceeded the axis limit, and the maximum value is indicated within brackets.

Next, we sought to investigate whether virtual spectratyping applied to single *IGHV* genes or families could reveal abnormalities of CDR-H3 length in patients vs. controls. Distribution of CDR-H3 length for the abundantly used *IGHV1-18* revealed deviations from the bell-shaped curve in patients W3, W4, and W7 as compared to controls (Figure 12A). Abnormalities of CDR-H3 length in patients with WAS were even more obvious for the *IGHV5* family (Figure 12B) and for the *IGHV3-66* gene (Figure 12C), which were less frequently used in WAS patients. Of note, abnormal distribution of CDR-H3 length was documented also among unique sequences, indicating restricted diversity of B lymphocytes expressing these genes.

**Figure 13:**
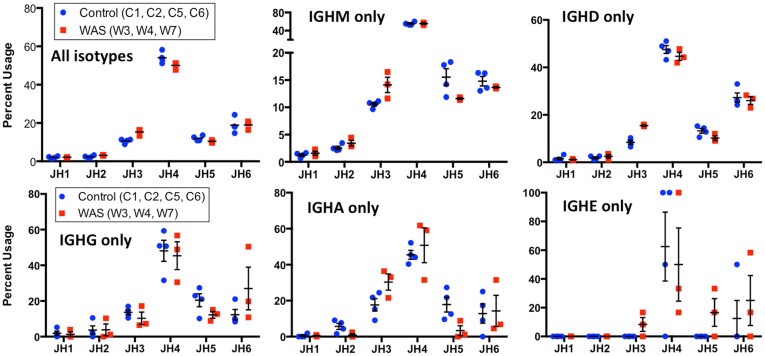
**Usage of *IGHC* genes in unique *IGH* sequences from WAS patients and controls**. Usage of *IGHC* families is displayed as the percentage of the total number of unique clonotypes for both control (blue dots) and WAS patients (red squares). Results are shown for all isotypes and for individual isotypes. Bars indicate the mean and SE.

When usage of *IGHJ* gene families was analyzed for all unique sequences, an increased usage of *IGHJ3* was observed in WAS patients than in controls (Figure 13). This was also confirmed when *IGHM*- and *IGHD*-containing transcripts were considered, and a similar trend was observed for *IGHA*-expressing clonotypes. In contrast, usage of *IGHJ5* was reduced within *IGHA*-expressing clonotypes from patients with WAS, and a similar trend was observed also for *IGHM*- and *IGHG*-expressing unique sequences (Figure 13).

**Figure 14:**
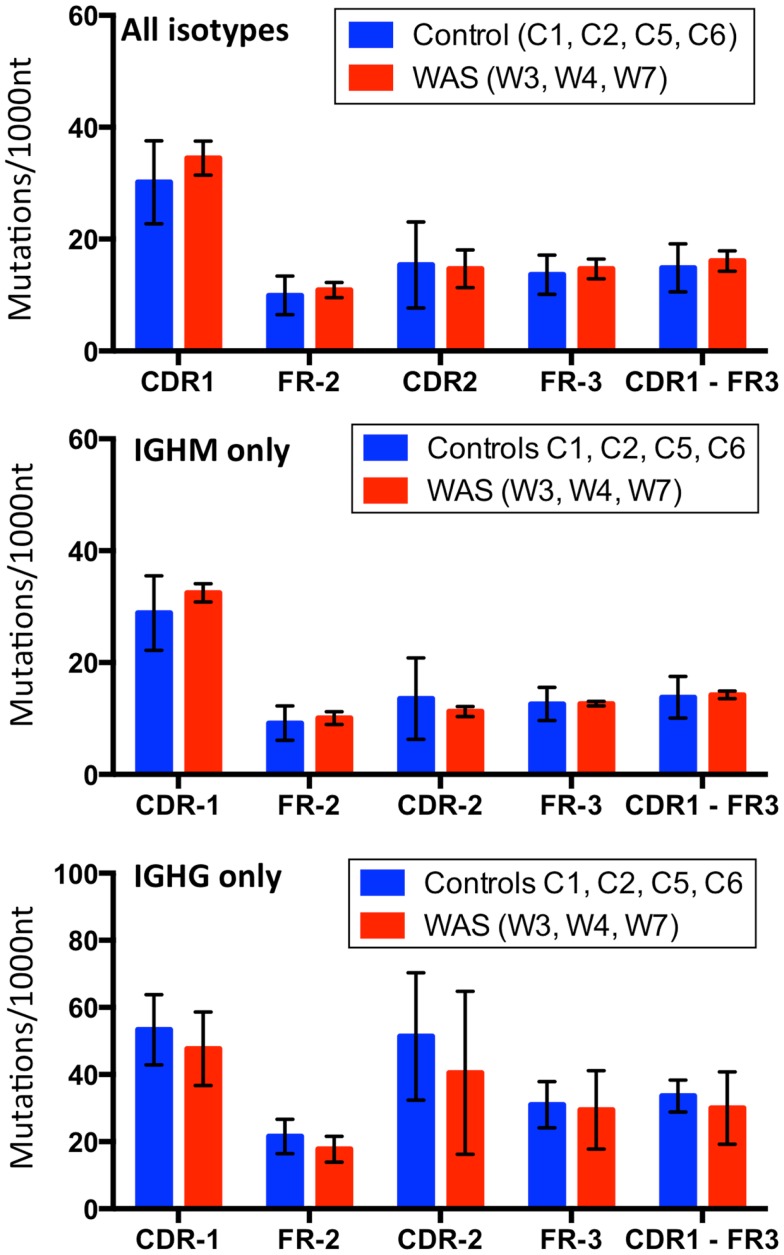
**Analysis of somatic hypermutation**. The rate (per 1,000 nucleotides) of somatic hypermutation in complementarity determining regions (CDR) and framework regions (FR) is shown for controls and WAS patients. Bars indicate the mean ± SD.

Next, we assessed the frequency and distribution of somatic mutations within CDR1, CDR2, and FR 2 and 3 for the total B cell population as well as within *IGHM*- and *IGHG*-expressing sequences. Overall, the rate and distribution of somatic mutations were not significantly different in patients and controls (Figure 14). However, when the analysis was restricted to *IGHG*-containing transcripts, there was a trend toward reduced mutation frequency in the CDR1 and CDR2 regions in WAS patients as compared to controls.

## Discussion

Development of NGS techniques has permitted analysis of the immune repertoire in healthy status and in various pathologic conditions to a depth that could not be achieved with previously available techniques. In the field of primary immunodeficiencies, NGS analysis of the antigen receptor repertoire has been limited so far to patients with leaky variants of SCID, in particular *RAG* defects (29–31). In this manuscript, we have reported for the first time on the use of NGS to analyze the T and B cell repertoire in patients with WAS. It had been initially thought that WAS is characterized by progressive T cell lymphopenia (32). Consistent with this, Wada et al. had demonstrated skewing of the *TRB* repertoire in patients older than 15 years of age, but not in younger patients (20). In contrast, Park et al. had found that T cell lymphopenia is common also in young WAS patients, and affects predominantly the naïve T cell compartment (10). More recently, Braun et al. have shown that indeed also young patients with WAS often have a perturbed TCR repertoire profile, with oligoclonal expansions of Vβ-expressing clonotypes (21). Here, we have confirmed and extended these observations. In particular, our data indicate that patients with WAS have a skewed *TRB* repertoire, with abundance of clonotypic expansions especially within memory CD4^+^ cells and in the entire CD8^+^ cell compartment. The vast majority of the patients studied were children, thus confirming that skewing of the *TRB* repertoire is a characteristic of WAS already early in life.

Several mechanisms may account for skewing of the *TRB* repertoire in patients with WAS. Because of the underlying immunodeficiency, patients with WAS are prone to recurrent infections, including chronic viral infections, which are often associated with expansion of effector memory T lymphocytes and CD8^+^ CD45RA^+^ CCR7^−^ T_EMRA_ cells (33). Seven of the eight patients studied here had a history of recurrent and/or chronic infections, and their immunological phenotype was characterized by increased proportion of effector memory and T_EMRA_ lymphocytes. Previous studies had demonstrated a higher degree of clonotypic expansions and reduced diversity of the *TRB* repertoire within memory than naïve T lymphocytes (34), possibly reflecting selective pressure conferred by antigenic stimuli. However, while recurrent and chronic infections may contribute to determine abnormalities of the *TRB* repertoire in patients with WAS, additional causal mechanisms must also be invoked.

Reduced, but detectable, WASp expression was demonstrated in patient W8, who maintained largely preserved *TRB* repertoire diversity. The clinical phenotype of this patient was less severe than in other patients reported in this study. Additional studies are needed to confirm whether partially preserved WASp expression and function, and less severe clinical phenotype, are also associated with maintenance of repertoire diversity.

Abnormalities of antigen receptor repertoire diversity and complexity have been demonstrated in various autoimmune diseases. This may reflect chronic self antigen-mediated stimulation or reduced plasticity of immune receptor generation in these patients (35, 36). However, it should be noted that none of the patients included in this study had clinically significant autoimmune disease.

Restoration of WASp expression by somatic reversion has been associated with partial or full rescue of TCR-mediated signaling (37), and may therefore, lead to emergence of clonotypic expansion of WASp-expressing cells. In this study, all three patients with somatic reversion showed uneven distribution of TRB clonotypes within T cell subsets in which somatic reversion was demonstrated (CD8^+^ lymphocytes from patients W1, W3, and W6; CD4^+^ lymphocytes in patient W6). On the other hand, a previous study demonstrated that revertant WASp-expressing cells from a single patient showed broader TRB repertoire diversity than WASp-cells from the same patient (37). The apparent contradiction between this observation and our data may be reconciled if reduced TCR signaling in WASp^−^ cells leads to impaired cell survival, and progressive reduction of repertoire diversity.

Indeed, our data clearly suggest that skewing of *TRB* repertoire diversity in WAS is not solely due to factors (chronic infections and somatic reversion) that sustain clonotypic expansions. In particular, use of NGS allows analysis of repertoire diversity and complexity also at the level of unique clonotypes. In normal individuals, generation of a broad spectrum of antigen receptor specificities translates into a polyclonal repertoire of expressed unique sequences, with a bell-shaped pattern of the distribution of CDR3 lengths demonstrated by virtual spectratyping. In contrast, we have observed increased usage of otherwise rarely utilized *TRBV* genes (and D-distal genes in particular), and abnormal distribution of CDR-B3 length among unique clonotypes expressed by CD8^+^ lymphocytes from patient with WAS. These data indicate for the first time that generation and/or maintenance of a diversified repertoire of CD8^+^ cells is compromised in patients with WAS. Impairment of thymic output in patients with WAS had been previously postulated (10); however, the possibility that reduced survival of peripheral CD8^+^ cells may also lead to progressive reduction of repertoire diversity cannot be excluded. Longitudinal studies, performed on uninfected patients identified at birth because of positive family history or of bleeding episodes associated with thrombocytopenia, may help dissect the role of impaired thymic output vs. reduced peripheral cell survival in determining skewing of the *TRB* repertoire.

Abnormalities of the B cell compartment in WAS include B cell lymphopenia, reduced number of CD21/CD35-expressing B cells and of unswitched and switched CD27^+^ memory B cells, and an increased proportion of circulating CD19^+^ CD21^low^ CD38^low^ autoreactive-prone B cells (16, 17, 38). Moreover, WASp-deficient B cells have increased signaling through the BCR and via toll-like receptors (13, 14), and this may trigger production of autoantibodies. Limited information is available on B cell repertoire diversity in patients with WAS. Two groups have recently reported skewed usage of *IGHV* genes and reduced rate of SHM in circulating B cells from patients with WAS (16, 17). However, only *VH3* and *VH4*-expressing clonotypes were included in these studies, and the method (cloning and sequencing) used allowed only for a limited number of sequences to be analyzed. By using NGS, we have confirmed skewing of the B cell *IGH* repertoire in three patients with WAS with presence of clonotypic expansions. Moreover, one of the three patients tested (W7) had an aberrant distribution of CDR-H3 length. Although usage of *IGHV* genes was relatively preserved, we have detected decreased usage of *VH3-66* within total sequences compared with controls. Castiello et al. have recently reported increased usage of *VH3-30* and *VH4-34* genes in WAS patients compared with controls (16), and increased usage of *VH4-34*, among both Cμ-expressing transitional B cells and CD21^lo^ CD38^lo^ B cells has been also reported (17). The *VH4-34* gene encodes for self-reactive cold agglutinin antibodies (39, 40), whereas *VH3-30* is highly represented among anti-platelet antibodies (41, 42). In this study, WAS patients and controls did not differ for the frequency of *VH3-30* usage, and increased usage of *VH4-34* was detected in one patient only (W4). However, none of the patients included in this study had clinical evidence of autoimmunity, and this may explain the lack of increased usage of *IGHV* genes associated with autoimmunity.

Although usage of *IGHV* genes was not significantly different in WAS patients vs. controls, virtual spectratyping demonstrated important abnormalities of CDR-H3 length distribution in WAS patients vs. controls. Of note, such abnormalities were also documented when analyzing CDR-H3 length of unique *IGH* expressed sequences. Castiello et al. have recently demonstrated increased release of bone marrow transitional B cells in WAS, secondary to decreased signaling through CXCL12 (16). Accelerated release of transitional B cells, whose repertoire is enriched for self-reactive specificities, may contribute to skewing of distribution of CDR-H3 length among expressed unique *IGH* clonotypes. However, the extent of such skewing is such that it is likely that reduced peripheral B cell survival may also play a role, similar to what discussed for T cells above.

WAS patients have increased serum levels of IgG, IgD, and IgE, and their IgM serum levels are often low (43). Consistent with this, we found the WAS *IGH* repertoire to have lower frequency of *IGHM*-containing sequences and increased frequency of *IGHD* and *IGHG*-expressing sequences, the lattermost being statistically significant. Despite all of our patients having elevated peripheral IgE levels and a history of eczema, the frequency of *IGHE*-expressing sequences was not different than in controls. This may reflect the observation that with the exception of patients W3 and W6, all other patients had only modest elevation of serum IgE. It is also possible that *IGHE*-expressing B cells are predominantly residing in tissues rather than in peripheral blood. Finally, it is important to keep in mind that IgE molecules account for a striking minority of all serum immunoglobulin molecules even in patients with a hyper-IgE phenotype, and larger sample sizes may be required to detect differences in the frequency of IGHE-containing transcripts.

Simon et al. have reported reduced rate of SHM among *VH3* and *VH4*-containing IGHG transcripts in sorted memory B cells from patients with WAS (17). Similarly, Castiello et al. have observed reduced rate of SHM within the V region of Cγ and Cα-containing *IGH* transcripts expressing *IGHV3* and *IGHV4* families (16). In the present study, the mean mutational rate of the V region (CDR1-FR3) of Cγ-containing transcripts was lower in patients with WAS than in controls, but the difference did not reach statistical significance, also because of the limited sample size. Finally, we observed a higher mutational rate in CDR than in FR regions, suggestive of *in vivo* antigen-driven selection (44).

In summary, by using NGS, we have demonstrated that the immune repertoire of WAS patients is characterized by clonotypic expansion and skewing of *TRBV* gene usage in memory CD4^+^ and total and memory CD8^+^ cells as well as skewing of *IGHV*, *IGHJ*, and *IGHC* usage in peripheral blood B cells. We have also shown that clonotypic expansions were especially prominent in patients with chronic infections and/or somatic reversion. Moreover, taking advantage of the analytical power of NGS, we have demonstrated that abnormalities of CDR-B3 and CDR-H3 length are also present among unique expressed clonotypes, suggesting inability to sustain generation or maintenance of a diversified repertoire. This adds a novel aspect to the complexity of the immune deficiency of this disease. Additional studies are needed to define diversity of the antigen receptor repertoire in other cell subpopulations that have been shown to be affected by the disease, such as regulatory T cells, unswitched memory B cells, and CD21^low^ CD38^low^ B cells. It will also be important to use NGS to investigate the antigen receptor repertoire in a larger number of WAS patients, including those with autoimmunity, in order to assess whether additional abnormalities may be detected. Finally, along with recently published data (16, 17, 21), this study may provide a framework to monitor WAS disease correction with HCT and gene therapy.

## Conflict of Interest Statement

The authors declare that the research was conducted in the absence of any commercial or financial relationships that could be construed as a potential conflict of interest.
